# The development of service user‐led recommendations for health and social care services on leaving hospital with memory loss or dementia – the SHARED study

**DOI:** 10.1111/hex.12477

**Published:** 2016-07-08

**Authors:** Carole Mockford, Kate Seers, Matt Murray, Jan Oyebode, Rosemary Clarke, Sophie Staniszewska, Rashida Suleman, Sue Boex, Yvonne Diment, Richard Grant, Jim Leach, Uma Sharma

**Affiliations:** ^1^ Division of Health SciencesWarwick Medical SchoolUniversity of WarwickCoventryUK; ^2^ Alzheimer's SocietyLondonUK; ^3^ School of Dementia StudiesUniversity of BradfordBradfordUK; ^4^ University/Users Teaching and Research Action Partnership (UNTRAP)University of WarwickCoventryUK; ^5^ Alzheimer's Society Research NetworkLondonUK; ^6^ National Institute for Health Research, Collaboration for Leadership in Applied Heath Research and Care, West Midlands (NIHR CLAHRC WM)CoventryUK; ^7^ Patient and Public Action GroupClinical Research NetworkCoventryUK

**Keywords:** dementia, hospital discharge, lay co‐researchers, memory loss, service provision, service user‐led recommendations

## Abstract

**Background:**

Health and social care services are under strain providing care in the community particularly at hospital discharge. Patient and carer experiences can inform and shape services.

**Objective:**

To develop service user‐led recommendations enabling smooth transition for people living with memory loss from acute hospital to community.

**Design:**

Lead and co‐researchers conducted semi‐structured interviews with 15 pairs of carers and patients with memory loss at discharge, 6 and 12 weeks post‐discharge and one semi‐structured interview with health and social care professionals and Admiral Nurses. Framework analysis was guided by co‐researchers. Two focus groups of study participants, facilitated by co‐researchers, met to shape and finalize recommendations.

**Setting and participants:**

Recruitment took place in acute hospitals in two National Health Service (NHS) Trusts in England. Patients were aged 65 and over, with memory loss, an in‐patient for at least 1 week returning to the community, who had a carer consenting to be in the study.

**Results:**

Poor delivery of services caused considerable stress to some study families living with memory loss. Three key recommendations included a need for a written, mutually agreed discharge plan, a named coordinator of services, and improved domiciliary care services.

**Discussion and conclusions:**

Vulnerable patients with memory loss find coming out of hospital after an extended period a stressful experience. The SHARED study contributes to understanding the hospital discharge process through the eyes of the patient and carer living with memory loss and has the potential to contribute to more efficient use of resources and to improving health outcomes in communities.

## Introduction

Having memory loss or dementia is not normally the reason people end up in hospital yet at least one in three hospital beds in elderly care, or acute medical wards are estimated to be occupied by people with dementia.^1^ A Dementia Hospital Research (DEMHOS) report^1^ indicated that people living with dementia who are admitted to hospital are mostly admitted to general medical and surgical wards with a range of acute physical conditions.

Hospitals can also identify those who are ‘at risk’ when returning home, or who will need extra help for the short or longer term. This, however, can also delay the discharge of the patient whilst a care package is organized.

Unnecessary long‐term stays in hospital are neither desirable nor helpful to the patient who is more susceptible to hospital borne infections or deteriorating physical and mental health. Health‐care services are put under pressure due to lack of beds for new patients, and social care services face fines over delayed discharges in relation to services and are obliged to reimburse the NHS for unnecessary extended days in hospital.^2^

Many patients return to their own homes with the consequence that care for people living with memory loss in the community is becoming an increasing pressure for the social care sector already under strain through insufficient funding.^3^

There is little published literature on the experience of discharge from hospital for patients and carers living with undiagnosed memory problems, or diagnosed dementia.^4^ It has been reported that the voices of those with dementia are seldom heard.^5^ The value of the involvement of lay co‐researchers has been documented particularly where the co‐researcher shares similar attributes, for example age or ethnicity^6^ or shares experiences akin to those being researched^7^ and who may be in a good position to access seldom heard groups.^8^ It has also been reported that lay researchers may be able to identify issues not readily recognized by professional researchers and create more meaningful interpretations from data.^9^

The aim of this study was to develop service user‐led recommendations to enable smooth transition for people living with memory loss from acute hospital to the community which will be disseminated to health and social care professionals involved in hospital discharge planning.

The objectives are as follows: (i) to explore the experiences of carers and people living with memory loss of service provision from hospital discharge, at 6 and 12 weeks post‐discharge;

(ii) to ascertain the involvement of carers and people living with memory loss in decision‐making around service provision at, and after, hospital discharge.

The SHARED study (Services after Hospital: Action to develop REcommenDations) involved lay co‐researchers in the collection and analysis of interview data from carers and patients who were discharged from an acute hospital, and from health and social care professionals involved in discharge.

REC approval: NRES committee London: Camberwell St Giles 14 LO 05/01.

## Methods

Less than 50%^10^ of people living dementia in England receive a diagnosis. The Research Ethics Committee advised widening the inclusion criteria from dementia to include those with undiagnosed memory loss.

### Lay co‐researchers

Twelve lay co‐researchers were recruited via local volunteer networks. The recruitment call requested that people who applied were current or past carers, have some experience of dementia, or be people living with undiagnosed memory loss or dementia. Three days of formal training were provided by the University in: research ethics and confidentiality with a general session on research methods; interviewing and interviewing practice; data analysis and conducting focus groups. A study group was set up by the Alzheimer's Society who oversaw the background and security checks and provided insurance cover for the co‐researchers.^11^

### Sample population

#### Patients and carers

Recruitment took place in acute hospitals situated in two NHS Trusts in central and South East England.

Research nurses were asked to recruit up to 15 pairs of patients and carers from each Trust from hospital wards just prior to discharge, who fitted the following criteria:

The patient:

was aged 65 and over;
had been an in‐patient for at least 1 week and was being discharged to their own home;
had memory loss possibly due to dementia, but not delirium or learning difficulties;
had a carer (family or friend) in close contact who also consented to be in the study or a personal consultee who could provide their opinion of whether the patient would want to contribute to the study, if the patient did not have the capacity to give personal consent.

It was important to the study that the patient was able to voice their experience where they had the capacity to do so, and on‐going process consent was gained prior to each interview^12^ in compliance with the Mental Capacity Act.^13^

Research nurses in sites 1 and 2 found it difficult to recruit from this population, they approached 30–35 patients in each site after pre‐screening for memory loss with ward staff from 12 and seven wards, respectively. The recruitment period was extended by 2–6 months overall. The research nurses reported that common reasons for not recruiting were that the patient: did not want to be involved in research, did not want anyone coming to their home, did not have a study partner (carer), that the research nurse could not contact the study partner or the discharge destination changed (e.g. from home to community hospital).

Twenty pairs of study participants were recruited: 15 in the host NHS Trust (site 1) and 5 from the second NHS Trust (site 2). Five pairs of study participants withdrew before the first interview, four due to ill health of the patient or the carer and one who had misunderstood the purpose for the study and who wanted more direct help.

Semi‐structured interviews were conducted with the remaining 15 pairs of participants soon after discharge (T1), and again at 6 (T2) and 12 (T3) weeks post‐discharge. Carers and patients were offered the opportunity to be interviewed individually or in pairs, all chose to be interviewed in pairs. Examples of interview topics included their experience of discharge from hospital, services received before and after hospital and their experiences of them, the level of their involvement in decision‐making and how they anticipate their future needs. At T2 and T3, the topics also included, for example, changes in patient and carer needs and responsibilities at home, how these were addressed and by whom. Lay co‐researchers attended the interviews accompanied by the lead researcher, where this was not possible; for example, a co‐researcher was not available then the interview was conducted by the lead researcher. The lead researcher obtained on‐going consent from the patient prior to the start of the interview. Co‐ researchers were responsible for conducting the co‐structured interviews guided by an interview schedule, and for probing for further information if more detail was required. Their availability to interview varied but in the main they stayed with the same pair of study participants in T1, T2 and T3 (Table 1).

**Table 1:** Twelve lay co‐researchers (CR) involvement in the SHARED study

	Attended training sessions	Interviewed study participants	Contributed to the analysis	Facilitated focus groups	Dissemination by co‐authoring papers	Dissemination by presentation
CR1	Y	Y	Y	Y	Y	Y
CR2	Y	Y	Y	Y	Y	Y
CR3	Y	Y	Y	Y	Y	Y
CR4	Y	Y	Y	Y	Y	Y
CR5	Y	Y	Y	0	0	0
CR6	Y	Y	Y	Y	Y	Y
CR7	Y	Y	0	0	0	0
CR8	Y	0	0	0	0	0
CR9	Y	0	0	0	0	0
CR10	Y	0	0	0	0	0
CR11	Y	0	0	0	0	0
CR12	0	0	0	0	0	0

No one withdrew from the study but one patient died after T2 and the carer, who was a neighbour, did not continue due to health reasons. During the 12‐week period, all participants were requested to keep diaries about their experiences of all services received, experienced or not received but required. They were given either paper diaries or electronic diaries according to their preference. Only one carer chose the electronic method. Six carers kept written diaries. Completed diaries were collected at the T2 interview and new ones given for collection at T3. The diaries were transcribed and analysed together with interview data.

#### Health and social care staff

Seventeen staff members who were involved in hospital discharge were recruited from hospital sites in three NHS Trusts or from the community. They were recruited via research nurses, colleagues, snowballing, that is colleagues of staff participants, and ‘cold calling’ via email or telephone. One semi‐structured interview was conducted with each participant. Topics included an explanation of the process of hospital discharge and how this differed for a patient with memory loss, how they experienced challenges and successes in discharge, what they would like to change, and how they felt health and social care services worked together. A third NHS Trust was included in the study to gain the perspective of a small group of specialist dementia nurses (Admiral Nurses). These specialist nurses did not work in the hospitals or community in sites 1 or 2 during the data collection period of this study but did provide a service in site 1 some months later.

All interviews were digitally recorded and transcribed verbatim.

### Analysis

Framework analysis was utilized, consisting of three stages: data management – which includes familiarization with the data, identification of emergent themes and categories, and developing a code matrix; descriptive accounts – where association is made between the themes and more abstract concepts are developed; and explanatory accounts – where meaning is found by reflecting on the previous two stages, keeping the interpretation true to the data, and applying wider application of the findings.^14^ This approach produces an effective and transparent trail leading back to the original data, thus demonstrating the rigour of the data analysis and the trustworthiness of the findings.^15^ There is little published literature describing the process of co‐analysis of data with co‐researchers and few examples on how this process can be performed.^16^ Pinfold *et al*.^17^ suggest a flexibility in approach if involving other stakeholders.

#### Data management

All interviews and diaries were transcribed with identifying features removed. Five lay co‐researchers, all of whom had participated in data analysis training, were each given one T1 anonymous transcript to read in‐depth prior to a 1 day group analysis meeting with the lead researcher. The co‐researchers took it in turns to raise the issues they felt were emerging from the transcript they had read. These items were recorded on a flip chart. They then read and discussed a further two transcripts each, totalling 15 transcripts from T1 comparing and contrasting items to earlier discussions and broadly grouping the items under descriptive headings, that is ‘carer experience’, ‘patient experience’, ‘(paid) carer organizations’, ‘health and social care services’, ‘other professions’, ‘communication’, ‘expectations’, ‘positives’, ‘general’. Everyone was given enough time to talk and they took it in turns to lead the discussion.

These themes emerged from the data with supporting categories, for example under ‘(paid) carer organizations’ came ‘issues with care workers’ and ‘time spent with patient’. This formed the framework for an in‐depth analysis of the interview data by the lead researcher. Facilitated by NVivo v10 the lead researcher analysed the interview data for T1 and T2 using framework matrices. Diary transcripts provided information concerning day‐to‐day activities and some personal descriptions of feelings towards caring and the person cared for. Data were incorporated into the analysis. T3 data were compared to T1 and T2 and in the main demonstrated a ‘settling down’ after hospital discharge, most of the difficulties for families lay in the first 6 weeks post‐discharge.

A summary analysis in a Word document was fed back to the co‐researchers by email and a further face‐to‐face meeting took place. Sixteen key statements relating to unmet need were agreed upon by reflecting on the descriptive findings. These were later reduced to 12 after allowing for repeats or similarities (Table 2).

**Table 2:** Twelve statements which formed the basis for recommendations

	Short heading	Statement	Subcomponents
1	To work in partnership	At hospital discharge, the patient living with memory loss, carer and services work in partnership:	By balancing skills, personal knowledge and time By allowing for life adjustments to be made so that there is a smooth, safe, transition from hospital to home By putting the patient and carer at the forefront of their care
2	To tailor and regularly review the discharge and care plan	Patients with memory loss, carers and services can regularly review the discharge and care plan so that it:	Accurately reflects personal and fluctuating circumstances, including readmission to hospital Provides the best and most suitable care environment for the patient Provides on‐going emotional support and advice to the carer which addresses their concerns
3	To have a written and mutually agreed discharge plan	Patients living with memory loss and carers should:	Clearly be made aware of the choices available to them at hospital discharge and beyond Have an initial written plan, which includes both health and social care information Be part of the agreement
4	To have timely information on planning of services, for example electronically	Patients with memory loss and carers should have a smooth transition from hospital to home and from secondary to primary care, examples are:	By having up to date information websites Timely information is provided electronically which acknowledges and recommends the next agreed step in the care plan
5	To have a named co‐ordinator of services and support	At hospital *admission*, a named co‐ordinator should be allocated:	To guide and support the carer and patient with memory loss through the health and social care system Be available for feedback and further information
6	To be informed about the implications and costs of care at home, respite care and care homes	Patients with memory loss and carers should be able to easily access information:	About the implications and costs of supporting a person at home On how to plan uptake of respite care and care homes if required
7	To have specialized support and signposting now and in the future	Specialized support, advice and signposting for carers and people living with memory loss and just out of hospital should be easily available:	To explain what health issues they may expect immediately To help them adapt to a changing life style
8	For the carer to have information on health status on the patient, and information on the availability of support in the community	Carers need to be informed about:	The health status and needs of the patient living with memory loss on leaving hospital to return home The availability, and choice, of services and support in the community
9	To have appropriately trained care workers	Patients living with memory loss and their carers should be assured:	The care package organized by the hospital with care agencies offers appropriately trained staff who work with the carer to provide a safe, reliable and patient‐centred service
10	To have more flexible care packages	Care packages need more flexibility to allow for:	The patient's recovery, for example to stay in bed longer than normal or to be taken out Being able to cancel and reinstate visits without risking the whole package or part of it being cancelled
11	To have improved care worker time spent with patient	Care worker visits to people living with memory loss need to be:	At the time agreed Offer good quality care Offer stability Offer meaningful social interaction
12	To have improved direct communication between families and care agencies	Carers and patients living with memory loss need:	An improved, direct form of communication with the care agencies regarding the type and quality of work conducted by care workers To ensure that the care provided meets the patient's needs, in particular for those who are new to the care system.

### Focus group using the nominal group technique^18^

All of the study participants (patients and carers) were invited to attend a focus group and received remuneration for their time and travel. Eight participants, including those with memory loss, agreed to participate, but only five were able to attend on the day. One person, who could not attend, contributed by mail (in total, there were five carers and one patient). They were asked to discuss each of the 12 statements in three blocks of four statements. They did this by breaking into two groups facilitated by two co‐researchers and asked to comment on, agree or disagree with the statements. The discussion was divided into three half‐hour sessions with allocated time for the two groups to reconvene to actively discuss and agree any anomalies. A moderator, not connected to the study, aided the process.

At the end of each session, the study participants scored each set of four statements from 0 to 100 (0 = of no importance; 100 = of great importance). The scores were averaged when all scores were received (including the one received by mail). This gave an indication of the value of importance placed on each of the statements. A wide variance was offered in order to explore the differences attributed to the order of importance of the 12 statements which may not be so visible in a range of one to ten, for example.

### Health and social care study participants

Health and social care staff who were study participants were contacted by email and asked to provide feedback to the 12 statements. Once the feedback had been received, the patient/carer study participants were asked to attend a second focus group meeting and feedback to the statements was discussed.

## Results

Twelve statements emerged from the descriptive account of patient/carer interviews, and these had converged into four clear key areas:

1. At discharge: problems emerged which included not being involved in discharge planning; confusion over what was agreed; confusion about changing the care package, not knowing what was happening.

Many families felt left out of the discharge planning and decision‐making process and that staff did not always understand their situation. This was reflected in some staff interviews which revealed that families were made to fit the system rather than the other way around. Carers also felt that they could only assimilate some information at the time of discharge as they were keen to return home.

It's not sort of… I don't know, you feel like you're having to go and ask them all the time because you're not being informed, and you feel like you're bothering them most of the time, […] I just think it would be better if there was a bit more information T1 015 (carer)

At home: problems focused on seeing too many professionals with no one person co‐ordinating the visits. Families in this study had reported being confused about who visits them and why, and who they should contact with questions.

But I think it's the sort of situation that if you're a carer like myself, you could get very aggravated by it because there are so many people with their fingers in the pie […] you get onto one person and then you have to get onto another. T3 053 (carer)

Carers may not be kept fully informed if they do not hold power of attorney.

Life with illness is so confusing when you are dealing with the medical profession. All we want is someone to be brave enough to tell us the Truth. We want to/need to know what we are dealing with. How ill is our Mum! T2 009 (carer) diary entry 08/12/14

At home: many expressed that they knew that information and support was available but they did not know if it was appropriate to their circumstances or how to access it. They did not know where to go to for help and advice about caring for the patient and did not know about availability of support in the community. Some carers worried about how to pay for the cost of social care at home in the future, and one very stressed carer described his inexperience of the system for getting urgent respite care which included six telephone calls with a care home and social services, a home visit and an assessment which resulted in a 6‐day wait for respite care

I rang up the care home […] She said I've got two [places] next week. I said, no, I don't want next week, I said, I want it today [Wednesday][…]. So by this time we'd rang Social Services and explained. But you have to go through a front desk and they ask you what you want, then they pass the message on and then the Social Services ring you back. […] So they rang back and they said, we've got to come out and assess her. Social Services said [she] can't come in until Monday. I said, okay, so that was it, so she went in on the Monday. T2 SN007 (carer)

Dementia advisors provided a service in one NHS Trust in the study, but study families were not informed of this service, with one carer finding a leaflet in his General Practitioner (GP) surgery by chance. Other services appeared difficult to find if the family did not know where to start looking or lacked the time to search for it.

Nobody's telling me. We got an internet connection now here […] otherwise it would be, you know, pretty grim trying to find anything. You get little booklets in the library and that was my only source. T1 052 (carer)

Daily care: carers experienced considerable stress from unreliable and inexperienced care workers, the inflexibility of care packages and losing existing care packages on readmission to hospital which had to be rearranged on discharge. There were examples of the lack of meaningful interaction with the patient, and the lack of good communication with the care agencies.

Late, rushed or missed care worker visits were reported, very little time to interact with the person they were caring for, and poor care standards. Many carers found it difficult to trust the service, with some doubting that care workers were adequately trained. There were examples of cancelled care packages with little or no warning, and difficulties in communicating with the care agencies:

I can't dance to their tune, you know, […] you're not communicating, you're not being consistent in the way that you're delivering the service, […] and I just felt it was a drastic move but I just felt we've just got to stop for both of our sakes, because it was making me ill. T2 052 (carer)

### Focus group 1

Facilitated by the lay researchers and moderated by a colleague not connected to the study, the study participants enthusiastically participated in the focus group. Much discussion focused on sharing personal examples of service provision which had been triggered by reading the statements. They discussed and agreed the 12 statements but made some changes to the wording; for example, statement 1 was considered too ‘wordy’.

### Ranking the statements

The study participants scored the 12 statements during the discussions at the focus group, and at the end of the meeting, they re‐visited their scores and changed them if they wanted to (Table 3). Some of the respondents scored 100 for every statement as of equal relevance and importance throughout the whole process of hospital discharge planning and care planning. The top ranking statements included those concerning the quality of home (domiciliary) care, having a written and mutually agreed discharge plan, and having a named co‐ordinator.

**Table 3:** Ranking of the 12 statements

Score/100	Twelve statements
97.5	To have improved care worker time spent with patient
96.7	To have more flexible care packages
95	To have appropriately trained care workers
95	To have a written and mutually agreed discharge plan
95	To have a named co‐ordinator of services and support
94.2	To be informed about the implications and costs of care at home, respite care and care homes
93.4	To have improved direct communication between families and care agencies
91.7	To work in partnership
91.7	Specialized support and signposting now and in the future
91.7	For carer to have information on health status of the patient and be informed of the availability of support in the community
90	To tailor and regularly review the discharge and care plan
85	To have timely information on provision of services, for example electronically

### Health and social care professional feedback

Seven of the 17 staff who were interviewed offered to comment, anonymously, on the 12 statements. Three sets of comments were returned by post and one telephoned with comments. Their comments illustrated their understanding of the challenges faced by carers and patients but also illustrated the difficulties they faced in changing ways in which they worked. For example, when asking about a written discharge plan, arguments against doing this included the extra work involved and that health‐care and social care services operated with separate electronic systems and that this could lead to misinformation. One member of staff argued that social workers in that NHS Trust did offer a written discharge plan, but no other profession was placed to do this. It was clear, in this Trust that the patient would have to have a social worker in order to have a written discharge plan.

### Focus group 2 – finalizing the recommendations

Once all the feedback was returned, it was collated and the 12 statements were incorporated into three overarching recommendations with subcomponents guided by the ranked importance of statements from focus group 1. This was presented to the study participants at focus group 2. Further discussions took place between the lay co‐researchers and the study participants. The recommendations were finally agreed with some amendments to the wording; for example, it was felt that a written and mutually agreed discharge plan should also be meaningful to the patient and carer, that is, patient‐centred.

The final recommendations are:

### Recommendation 1

To have a written, mutually agreed and meaningful discharge plan.

This must include carers, patients, health and social care personnel working together to put a discharge plan in place which is quickly followed up by a short‐term or long‐term care plan as needed. They would be working in partnership and tailoring and reviewing the plan together. A staff member would provide timely information or updates by phone or electronically (email or text message).

### Recommendation 2

To have a named co‐ordinator who is a point of contact for services and support.

This role would be dual purpose: (i) to be allocated at admission or soon after, where there is a known undiagnosed memory problem or diagnosed dementia, whose purpose is to meet the patient and carer, and to guide them through the health and social care process from discharge back to the community; (ii) to provide information when it is needed, such as that on costs of care at home, respite and care homes; to signpost to community services; and to signpost to specialist support for carer and patient.

### Recommendation 3

To improve the quality of care provided by care agencies in patients’ homes.

This would include improved quality of care worker time spent with the patient particularly social interaction; more flexible care packages; appropriately trained care workers; and improved communication with the homecare agencies.

## Discussion

The recommendations developed from this study reflect the experience of *receiving* services post‐hospital discharge by people who live with undiagnosed memory loss or dementia. The SHARED study has stressed the necessity to look more closely at how services are received by vulnerable populations such as those living with memory loss, particularly in the first 6 weeks post‐discharge. It also reminds us that families experience the discharge from hospital and settling back at home as a single event. Yet in contrast, this can trigger the involvement of multidisciplinary teams and agencies in the discharge process who may appear as a confusion of faces from many services which can put families under considerable stress. Previously, the Department of Health have recognized the need to involve patients and carers in all stages of discharge planning promoting communication, information as well as a suggestion for written information for patients.^19^ The Five Year Forward View (2014)^20^ for the NHS is working towards new models of integrated care and greater joint working by the health and social care services. This study demonstrates that there are still unmet needs in the system and that the process of a complex discharge and the setting up of a care plan may not be experienced by those receiving services as expected by those providing them particularly by those who have limited recall of events and no written evidence of discharge planning.

Findings from this study illustrate the difficulties experienced by families at hospital discharge who also have to cope with memory loss and emphasize the need to review the number of multi‐agencies, assessments and processes involved. The overriding message is that carers and patients feel left out of the very process that is meant to support them.

Some of the recommendations have raised known problems, and the Prime Minister's 2020 Challenge on Dementia 21 is seeking to address similar issues. Study participants were interviewed between July 2014 and January 2015 so any new local policies may not have been in effect during this time such as the Care Act^22^ coming into effect in April 2015 which, for example, legally obliges local councils to keep all carers informed and supported.

### A written, mutually agreed and meaningful discharge plan

The Department of Health recognize that up to 25% of people in hospital have dementia and they are committed to asking every hospital to become dementia‐friendly; however, very little is being focused on the discharge from hospital. The Prime Minister's 2020 Challenge on Dementia^21^ suggests that patients with dementia are discharged back to the community in a timely and appropriate way, but there are no suggestions on what this actually means.

In this study of two NHS Trusts, there appeared to be a lack of patient‐centredness and services were prescriptive; for example, a maximum of four care worker visits a day at key times with little flexibility. A written discharge plan could remind people living with memory loss, including carers, about what was agreed at a time when they may not have been able to comprehend everything which was happening. One that was mutually agreed and meaningful (patient‐centred) would help to smooth the process back to the community.

### Named co‐ordinator

From 1 April 2015^21^ patients with dementia will have access to a named GP who will have responsibility for the oversight of their care. GPs will have a leading role to ensure that people living with dementia have coordination and continuity of care. It is not clear how involved GPs will be at hospital discharge which will address the current concerns of patients and carers living with memory loss. The hospital discharge teams, in this study, consisted of a multitude of health and social care staff, none of which appeared to take on the role of a named co‐ordinator. There were no specialist nurses, that is Admiral Nurses, working in the community in these two NHS Trusts at the time of the data collection, which may explain why study participants did not know of the service they provide. The study included people with undiagnosed memory loss as well as those with diagnosed dementia, those with no diagnosis appeared to be outside of the loop for specialized support.

### Quality of home care

As data were collected post‐hospital discharge, much of the focus of carers and patients was on the delivery of social care and especially the work of homecare agencies. Reports from study participants indicated that daily care provided by homecare agencies was below expectations in the main, including in particular, the unreliability of timed visits, not working together with the carer, and the poor quality of care provided. Recent recommendations from the National Institute for Clinical Excellence (NICE) have brought attention to the urgent need to improve home care services.^23^ The 2020 Challenge on Dementia^21^ suggests that care providers improve the experience and care for patients and their carers at home and that evidence‐based training is provided for staff although the quality of training is not emphasized. By 2018, the government intends for care workers to be trained, be able to spot signs and symptoms of dementia and be able to signpost people to further support and care.

Future intended changes supported by the Prime Minister's 2020 Challenge on Dementia,^21^ the Five Year Forward View (2014)^20^ and NICE recommendations^23^) are moving in the right direction for the provision of better services. The experience of receiving services, however, can be quite different from the expectations of service providers and might not be reaching the very people they are intended to serve or are not of the quality people in the community expect. These service user‐led recommendations have identified the needs which are not being met by those who are the most vulnerable in our society. The third sector, for example charitable organizations, provides vital services not available from health and social care services, but many of our study participants were unaware of them or did not try to contact them. Those with a diagnosis of dementia may find it easier to link into specialized services, for example Alzheimer's Society or Dementia UK. Those with undiagnosed memory loss may not be accessing vital support, and this is an area which needs further investigation.

### Limitations of the study

Although hospital policies prefer patients to return to the community, many are re‐directed to another destination such as a community hospital and this was a major reason given by research nurses for people being excluded from recruitment into the study. This study included those who agreed to take part. It is not known if their experiences and views differ from those who did not take part in the study. Research evidence is taken from the perspective of the person living with memory loss and their carer who reported what did not work so well for them, we are not able to corroborate this or the circumstances in which events happened. However, the final three recommendations represent the key problem areas.

Study participants did not include those from Black, Asian and Minority Ethnic (BAME) groups. There is no obvious reason for this. There is a gap in research studies regarding the inclusion of BAME groups in dementia services. Some of these challenges may be very different from this study's participants, for example language, culture or stigma, warranting research in its own right.

The study participants shared positive experiences, and these were included in the analysis but are not reflected in the recommendations. These illustrated the kindness and caring attributes of many individual care workers.

## Conclusion

The three recommendations cover a multitude of service agencies. Implementation of each recommendation may be complex and require separate interventions in acute hospitals and in the community. The SHARED study contributes to the understanding of the hospital discharge process through the eyes of the patient and carer living with memory loss. It provides evidence about how services are received which may differ from a service provider's expectations. Results from the study provide valuable information for decision‐makers at all levels when considering services for patients with memory loss and their carers who are leaving hospital and returning home. The recommendations provide a starting point for planning and improving services and have the potential to contribute to more efficient use of resources and to improved health outcomes in communities.

## Conflict of interests

No conflict of interests have been declared.

## Source of funding

This study presents independent research funded by the National Institute for Health Research (Research for Patient Benefit (RfPB) programme: PB‐PG‐1112‐29064 – Carer and patient‐led development of recommendations for people with dementia returning home from hospital: understanding what is important). The views expressed in this publication are those of the authors and not necessarily those of the NHS, the National Institute for Health Research or the Department of Health.
